# MaxGRNet: A multi-axis vision transformer with improved generalization for eye disease classification using explainable AI with insertion-deletion operations on fundus images

**DOI:** 10.1371/journal.pone.0346329

**Published:** 2026-04-08

**Authors:** Md Mehedi Hasan Santo, Fuyad Hasan Bhoyan, Fuad Ibne Jashim Farhad, Fahmid Al Farid, Sovon Chakraborty, Md Humaion Kabir Mehedi, Jia Uddin, Hezerul bin Abdul Karim

**Affiliations:** 1 Department of Information Technology, Central Queensland University, Melbourne, Victoria, Australia; 2 Department of Computer Science and Engineering, University of Liberal Arts, Dhaka, Bangladesh; 3 Centre for Image and Vision Computing (CIVC), COE for Artificial Intelligence, Faculty of Artificial Intelligence and Engineering (FAIE), Multimedia University, Cyberjaya, Selangor, Malaysia; 4 Department of Computer Science, Old Dominion University Norfolk, Norfolk, Virginia, United States of America; 5 Department of Computer Science and Engineering, BRAC University, Dhaka, Bangladesh; 6 AI and Big Data Department, Endicott College, Woosong University, Daejeon, Republic of Korea; University of Donja Gorica, MONTENEGRO

## Abstract

Eye diseases, including diabetic retinopathy (DR), glaucoma, and cataracts, represent a major global health concern and can lead to severe visual impairment or blindness if not identified in a timely manner. This study proposes a novel eye disease classification framework based on a multi-axis vision transformer (MaxViT) applied to color fundus images with Explainable Artificial Intelligence (XAI) techniques to enhance model transparency. The proposed architecture integrates transformer-based attention mechanisms with Global Response Normalization (GRN)-based multi-layer perceptron (MLP) layers to capture complex spatial and contextual relationships within fundus images effectively. The model was evaluated on a publicly available eye disease classification dataset using a five-fold cross-validation strategy to assess its robustness and generalization. The experimental results show that the proposed approach consistently outperforms conventional Convolutional Neural Networks (CNNs) and Vision Transformer (ViT) variants, including ResNet50, Swin-T, MaxViT-T, and ViT-B16. The model achieved a macro-averaged test accuracy, precision, and recall values of 96.75%, 96.70%, and 96.80%, respectively, with paired statistical t-tests confirming that these improvements were statistically significant. Rigorous preprocessing techniques were employed to improve data consistency, and XAI-based visual explanations provided insights into the model’s decision-making process, supporting interpretability in ophthalmic image analysis. Overall, the proposed MaxViT-based framework is robust and computationally feasible for research-oriented evaluation approaches for automated fundus image classification, highlighting the potential of advanced transformer architectures for future decision-support and research-oriented ophthalmic applications.

## 1. Introduction

Artificial Intelligence (AI) methodologies, particularly transformer-based architectures, are increasingly essential in disease classification tasks and hold significant potential for detecting vision impairment [1]. Globally, over 2.3 billion individuals are affected by various ocular diseases, including glaucoma, diabetic retinopathy, and cataracts [2]. Current computer-aided solutions predominantly utilize fundus images to detect these conditions. These solutions often depend on handcrafted features that lack generalizability across diverse conditions [3]. Moreover, fundus images are among the most accessible imaging modalities for diagnosing eye diseases, requiring careful preprocessing and analysis [4]. In addition to disease detection, transparency in decision-making is crucial in the medical field. The processing of fundus images is both time-intensive and prone to inter-observer variability [5]. Convolutional neural network (CNN)-based architectures are primarily employed to process fundus images to facilitate early disease detection. However, a significant limitation of CNNs is their focus on local receptive fields, whereas understanding long-range relationships among retinal regions is imperative [6].

Our objective was to process fundus images and address the limitations of the current state-of-the-art architectures. Fundus images can indicate specific diseases, necessitating a framework capable of accurately detecting multiple eye diseases to alleviate the widespread burden among visually impaired individuals [7]. For instance, the global geometry of the optic disc is critical for glaucoma detection, whereas lesion distribution is vital for diabetic retinopathy. Additionally, retinal pathologies are often sparsely distributed in fundus images, and small lesions can be critical for diagnosis. The self-attention mechanism of the transformer model can selectively process clinically relevant areas [8]. Fundus images may also be noisy owing to variations in the illumination during processing. As CNNs focus solely on filters, improved generalization is required. Transformer architectures can model long-range dependencies across the entire image through self-attention mechanisms rather than relying solely on fixed convolutional filters [9].

In response to these limitations, the primary aim of this study was to develop a robust framework for multi-class eye disease classification using color fundus images. We propose a multi-axis transformer-based architecture designed to efficiently capture local and global image features. Following the experiment, we compared the results of this framework with the established architectures in this domain [10]. These findings highlight the potential of transformer-based models to advance medical imaging, particularly in managing eye diseases. To elucidate the decision-making process of the proposed architecture, we integrated Explainable Artificial Intelligence (XAI).

The main contributions of this study are as follows:

- A new hybrid architecture is introduced by replacing the standard multi-layer perceptron (MLP) layer in the base MaxViT-T model with a Global Response Normalization (GRN)-based MLP derived from the ConvNeXtV2 architecture, enabling improved global–local feature modeling.
- The proposed framework was extensively evaluated using multiple performance metrics and five-fold cross-validation, demonstrating consistent performance improvements over state-of-the-art architectures, including ViT-B16, Swin-T, ResNet50, and MaxViT-T.
- Robust preprocessing strategies were incorporated to enhance data consistency and model generalization across diverse fundus image conditions.
- Explainable artificial intelligence (XAI) techniques, including Grad-CAM and quantitative insertion–deletion analysis, are employed to improve model transparency and provide insights into the decision-making process.
- Comprehensive experimental and statistical analyses are conducted to assess predictive performance, robustness, and explainability, establishing the proposed framework as a reliable computational approach for ophthalmic fundus image analysis.

## 2. Background study

### 2.1 Literature review

Eye disease (ED) classification has become one of the most important research areas in both the medical and computer vision domains due to the critical need for early detection and prevention of severe vision impairment, including blindness. Researchers have utilized various deep learning models, particularly convolutional neural networks and transformer-based architectures, to enhance the accuracy of disease detection using fundus images. In this section, we have summarized the existing research experiments along with their limitations. Based on the limitations, we then turn our attention to proposing a novel framework that reflects improvement over the state-of-the-art architectures.

Image classification and its use in medical imaging depend heavily on convolutional neural networks. The utilization of fundus images is extensive for the classification of Diabetic Retinopathy (DR) using CNN architectures. Although CNN-based architectures have demonstrated high classification accuracy, they require large-scale datasets for effective training, and their substantial computational cost limits their widespread adoption in clinical settings. Furthermore, the authors [11] investigated CNN-based methodologies for detecting DR, Glaucoma (GLC), and Age-related Macular Degeneration (AMD), underscoring the importance of preprocessing steps such as contrast enhancement. Moreover, existing architectures reported in the literature often struggle to handle variations in image quality across diverse datasets, raising concerns regarding their generalization capability. In [12], authors proposed a CNN architecture that was capable of handling binary classes of data. The primary objective in medical diagnosis is to perform accurate multi-class classification, especially for eye disease classifications. To overcome these problems, statistical methods such as adaptive histogram equalization were employed in [13] to render fundus images properly. Under numerous imaging conditions, these statistical methods often fail to provide precise classification results.

Transformer-based models are increasingly gaining popularity over CNNs. The Vision Transformer (ViT) leverages self-attention mechanisms to model global contextual relationships within images, which is particularly important for medical imaging applications. In [14], a ViT + CSRA model was used on retinal images and outperformed conventional CNN models in accuracy and feature extraction. However, ViT models need large datasets for training, which is a challenge for smaller clinical datasets. To overcome this, [15] suggested a hybrid model combining MobileNet and DenseNet-121 with an Artificial Neural Network (ANN) classification head to reduce the need for large datasets while achieving high performance with accuracies of 93.4% and 94.1%. Preprocessing methods like Contrast-Limited Adaptive Histogram Equalization (CLAHE) and median filtering are often used to improve image quality in ED classification.

The integration of CLAHE improved the contrast in fundus images, which led to better performance of the classification model [16]. However, [17] said that CLAHE can add noise, so it requires additional screening methods to maintain picture quality while increasing contrast. Feature extraction is an important part of ED classification. In [18], a deep learning model was used to extract features from fundus images that could be used to classify different eye diseases. The model used a feature selection method that improved classification success, but it required extensive computing power to run. Similarly, [19] examined how feature fusion methods could be used to combine multiple features from different layers of the model, which would make glaucoma detection more accurate. In [20], a CNN-based model was proposed to classify retinal images from the STARE dataset, which consists of 15 categories of eye diseases. However, such architectures may exhibit reduced robustness under varying imaging conditions, and their computational cost can limit real-world deployment. The study utilizes ResNet50 and InceptionV3 to classify fundus images into three groups: normal, macular degeneration, and tessellated. After applying standard optimization procedures, the models achieved classification accuracies of 93.81% and 91.76%, respectively. Table 1 provides a detailed overview of the literature in this domain. The previous research gaps are also listed in this table.

**Table 1:** Identified research gaps from the literature review.

Articles	Architectures	Research Gaps
Topaloglu et al. [21]	CNN with Care model	Higher computation complexity with lower performance
Chea et al. [11]	ResNet50 and others Ensemble TL Architectures	Lack of performance metrics and evaluation strategies.
Bernabe et al. [12]	CNN with two layers of convolution	Only binary classification was implemented
Mayya et al. [13]	VGG-16 and others Ensemble TL Architectures	Higher resource cost
Gu et al. [14]	Vision Transformer and Residual Attention (ViT+CSRA)	Higher computation complexity with lower efficacy
Sarki et al. [16]	CNN with five layers of convolution	Only binary classification was implemented
Chea et al. [18]	SEnet with HRnet as Backbone	No advancement in DL architecture
Aranha et al. [19]	Modified CNN with VGG-16 as Backbone	Lack of performance metrics and evaluation strategies.
Pan et al. [22]	InceptionV3 and ResNet50	Proper validation of the data was missing and there was a lack of transparency
Gu et al. [23]	Hybrid Convolution and Vision transformer based model	Higher computation complexity with lower performance

A vision transformer-based mixed feature extraction method was developed in [23] to classify eye diseases. Multiple datasets showed better performance with the hybrid method, but it was too expensive for real-time clinical use. Furthermore, CNNs and ViT models have a lot of promise in ED classification, but they face problems such as the large size of the dataset, the unpredictability of the images, and the high cost of computing. The model performed better using preprocessing methods, feature extraction, and ensemble learning. Nevertheless, problems still exist regarding their real-time use and application to other situations. It is recommended that future studies focus on developing models that are more efficient and can handle smaller and more varied datasets while remaining highly accurate.

### 2.2 Dataset description

The Eye_diseases_classification dataset is publicly available [24] and was used as the primary dataset (DS1). It consists of retinal images that are classified into four categories namely normal, diabetic retinopathy, cataract, and glaucoma, and each category has approximately 1,000 images. These images were sourced from several esteemed databases, such as the Indian Diabetic Retinopathy Image Dataset (IDRiD) [25], Ocular Recognition [26], and High-Resolution Fundus (HRF) databases [27]. To ensure class balance and avoid bias during training, exactly 1,000 images per category were used, as summarized in Table 2. For DS1, the dataset was partitioned into training, validation, and test subsets using an 80:10:10 ratio. The training and validation subsets were used during model optimization, while the test subset was strictly reserved for final performance evaluation. No image-level overlap was permitted across these splits.

**Table 2:** Class distribution of the dataset (DS1).

Eye Diseases	The Number of Images
Cataract	1000
Diabetic Retinopathy (DR)	1000
Glaucoma	1000
Normal	1000
Total	4000

The “Eye Disease Image Dataset” published on Mendeley Data [28] was utilized as the secondary dataset (DS2) for independent evaluation and robustness analysis. Table 3 presents the distribution of the secondary dataset (DS2), which contains ten categories of ophthalmic diseases represented by both real and augmented samples. The original dataset exhibited substantial class imbalance. Therefore, the dataset was first partitioned into training (70%), validation (20%), and testing (10%) subsets. After partitioning, offline data augmentation was applied independently within each subset to balance the class distribution and increase the number of samples to 2,000 per category. For example, the Central Serous Chorioretinopathy (CSC) class originally contained 101 real images, which were supplemented with augmented samples to reach a total of 2,000 images. A similar augmentation strategy was applied to the remaining classes. Augmented images were generated only from samples within their respective subsets, ensuring that variants of the same original image were not distributed across different data splits and thereby preventing potential information leakage.

**Table 3:** Distribution of samples in dataset (DS2) for different diseases.

Class Name	Real Sample	Augmented Sample	Total Sample
Central Serous Chorioretinopathy	101	1899	2000
Diabetic Retinopathy	1509	491	2000
Disc Edema	127	1873	2000
Glaucoma	1349	651	2000
Healthy (Control)	1024	976	2000
Macular Scar	444	1556	2000
Myopia	500	1500	2000
Pterygium	17	1983	2000
Retinal Detachment	125	1875	2000
Retinitis Pigmentosa	139	1861	2000

Models were trained and evaluated separately on DS1 and DS2. DS2 was not used for training, validation, or hyperparameter tuning of models evaluated on DS1, and vice versa. Therefore, DS2 was not employed as an external validation dataset in a cross-dataset training setting; rather, it was used as an independent dataset to assess robustness under differing class distributions and image characteristics. It is important to note that DS1 consists exclusively of retinal fundus images, whereas DS2 includes a mixture of internal fundus images and certain external ocular images (e.g., pterygium), introducing additional heterogeneity.

**Fig 1:**
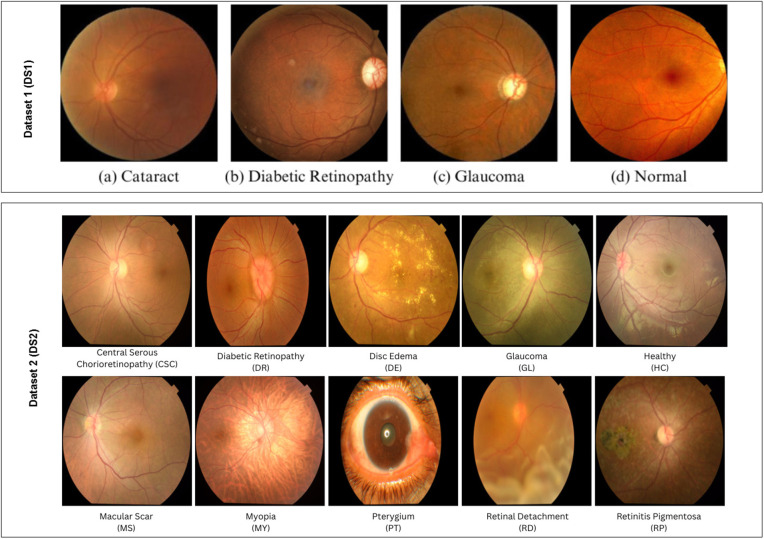
Sample images from Eye_Disease_Classification dataset: DS1 and color fundus images dataset: DS2.

Both DS1 and DS2 provide single-label annotations per image. Multi-label or overlapping disease annotations were not available in the datasets. Therefore, the classification task was formulated as a mutually exclusive multi-class problem. Sample images of the two datasets are shown in Fig 1.

### 2.3 Multi-axis vision transformer (MaxViT)

In recent years, there has been significant competition for iterative improvements based on convolutional neural networks and the relatively recent transformer architecture in terms of optimal performance for standard image vision tasks. In a paper published in ECCV 2022, researchers from Google Research and the University of Texas at Austin introduced MaxViT [29]. By addressing the issue of global attention in transformers, the multi-axis vision transformer aims to combine the advantageous features of both convolutional neural networks and transformers. MaxViT utilizes a technique known as multi-axis attention, which encompasses both block and grid attention. This approach enables MaxViT to address the global and local contexts with O(N) complexity. The final MaxViT architecture follows the typical hierarchical design of convolutional neural network practices (e.g., ResNet). Instead, it constructs a novel type of basic building block that unifies the MBConv and grid attention layers. MaxViT has demonstrated superior performance compared to all existing state-of-the-art algorithms for standard image tasks, such as object detection, image classification, and aesthetic assessment. Additionally, the model has demonstrated strong generative modeling abilities on ImageNet, indicating that MaxViT blocks have significant potential as a universal vision module.

## 3. Methodology

**Fig 2:**
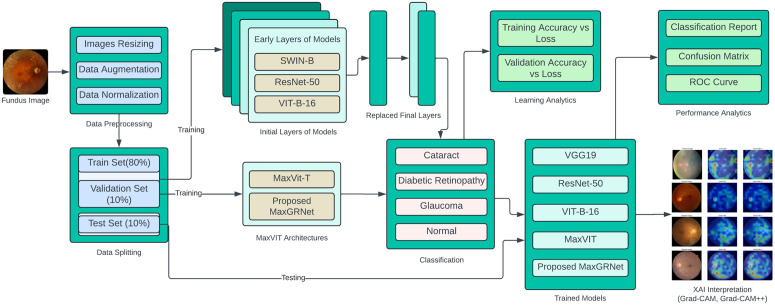
Complete block diagram of experiments to diagnose eye disease.

In this study, the authors identified the publicly available dataset containing eye fundus images. Comprehensive preprocessing techniques were applied to ensure optimal performance before utilizing these images in pre-trained models, including the MaxViT-T and MaxGRNet architectures. These models were trained and validated over 50 epochs using a batch size of 64. The experiments were conducted on Google Colab using the GPU L4. The methodology is presented in the following order: image preprocessing and augmentation, proposed MaxViT model, replacing MLP layer with GRN-based MLP layer, and evaluation strategies of the experiments. Fig 2 shows the proposed research methodology, in which MaxGRNet is the primary model. Four additional pre-trained backbone architectures Swin-T, ResNet50, ViT-B16, and MaxViT-T were incorporated as competitive baselines and fine-tuned under a consistent transfer learning framework to ensure a fair and rigorous comparison. Finally, the performances of these models were compared for the classification tasks.

### 3.1 Preprocessing and augmentation of the dataset

Enhancing and cleaning images is crucial in imaging to address challenges such as noise and distortions that can affect the efficacy of models in analyzing data and producing results.

The preprocessing phase began by standardizing the image sizes because the dataset contained images of varying dimensions. All images were standardized to a size of 224 x 224 pixels to ensure consistency in the model’s input data preparation. This step is crucial for enhancing the image quality and ensuring that the models are properly prepared for processing.

We applied image normalization to ensure consistent intensity values across all samples. Specifically, the transformation [mean=[0.485, 0.456, 0.406], std=[0.229, 0.224, 0.225] is applied.

Image augmentation techniques enhance the flexibility of the model and its capacity to identify patterns. Various techniques were applied, including random horizontal and vertical flips, color jittering, and Gaussian Blur. These enhanced samples expanded the training dataset, contributing to improved model performance on new data.

For DS1, augmentation was performed dynamically during training (on-the-fly) and applied exclusively to the training subset within each fold of the five-fold cross-validation framework. Augmentation was executed after dataset partitioning, ensuring that validation and test subsets remained untouched. No augmented samples were stored or reused across folds, thereby preventing information leakage.

For DS2, the dataset was first partitioned into training (70%), validation (20%), and testing (10%) subsets. After the split, offline data augmentation was applied independently to each subset to address class imbalance and ensure that all classes contained an equal number of samples (2,000 images per category). This augmentation process was performed separately within the training, validation, and testing sets to avoid data leakage between subsets.

### 3.2 Proposed MaxGRNet architecture

**Fig 3:**
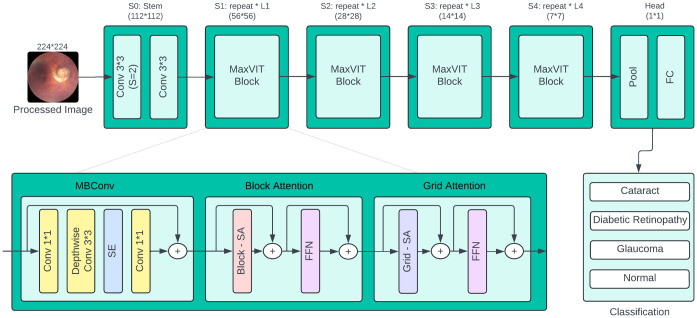
Architectural overview of multi-axis vision transformer (MaxViT-T).

The proposed system merges a GRN-based MLP with a multi-axis vision transformer (MaxViT) to process fundus images and diagnose a range of eye diseases, including cataracts, diabetic retinopathy, glaucoma, and normal conditions, as illustrated in Fig 3. The process begins with the feeding of the 224 x 224 pre-processed fundus image by a row of convolutional layers (Conv 3 x 3 and Conv 1 x 1), and a MaxViT block. This process is repeated several times in various stages (S1 to S4). The stages are a hierarchical refinement of the image features; the resolution and scale is successively decreased to obtain more abstract and global patterns of the image.

**Fig 4:**
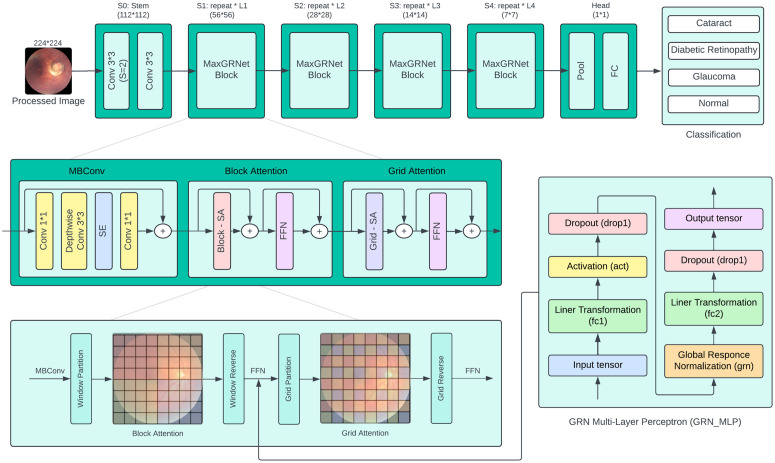
Architectural overview of proposed MaxGRNet with GRN_MLP integration.

The primary components of MaxViT blocks consist of MBConv layers along with block attention and grid attention mechanisms, as depicted in Fig 4. The design helps the architecture to capture both local and global image dependencies. MBConv layer enhances the local features with the help of depthwise convolution and Block Attention focuses on the areas of interest inside the image blocks. This is supplemented by the Grid Attention module, which looks at patterns on a bigger grid and enables multiscale feature extraction. The final stage applies global pooling to reduce the spatial dimensionality of the feature maps, followed by a fully connected (FC) layer that classifies the input into the corresponding number of disease categories (four for DS1 and ten for DS2).

**Fig 5:**
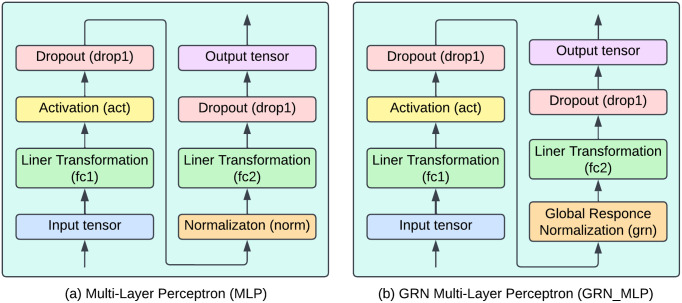
GRN-based MLP for proposed MaxViT architecture.

In the MaxViT framework, the multi-layer perceptron (MLP) layer was enhanced by incorporating the global response normalization (GRN) technique [30] to broaden the model’s applicability. Fig 5 depicts the structure of the MLP with GRN integration. The MLP, a vital element of artificial neural networks, is composed of interconnected neuron layers and is frequently employed in vision models like MLP-Mixer and vision transformers [31]. In deep learning, normalization methods are crucial for boosting generalization, stability, and training efficiency. These methods tackle issues such as vanishing or exploding gradients, improve gradient flow, and accelerate training convergence, thereby enhancing the overall generalization of models. The GRN method, introduced in the ConvNextV2 paper in 2023 [30], is designed to improve channel contrast and selectivity. The GRN handles an input feature tensor *X* with dimensions H×W×C through three primary phases: feature calibration, normalization, and global aggregation. This method is simple to implement, requiring only three lines of code as well as no learnable parameters. The GRN successfully addresses the drawbacks of the MaxViT architecture by improving its diversity of features and the quality of feature representations. It converts the maps of spatial features to the global functional vectors, normalizes the responses based upon summed up values, and modifies the input responses based on the calculated normalization scores. The process improves the quality of representation and increases the overall performance of the model.

### 3.3 Environmental setup and model selection

All models were run in the Google Colab environment. Table 4 provides a detailed overview of the hardware specifications and training configurations employed in the experiments. The training was performed using 50 epochs with a batch of 64 and the Adam optimizer. To ensure constant convergence and avoid overfitting, we chose the learning rate to be 0.00001 and weight decay 0.00003.

**Table 4:** Training configuration and hardware specifications.

Parameter	Value
Epochs	50
Batch Size	64
Optimizer	Adam
Learning Rate	0.00001
Weight Decay	0.00003
**Hardware**	**Specification**
GPU	Google Colab L4

We chose a wide range of deep learning models to be compared in order to evaluate performance in different paradigms of architecture in the most comprehensive way. This includes transformer-based architectures like ViT-B16, Swin-T, and MaxViT-T, alongside convolutional neural network backbones such as ResNet50. Additionally, the hybrid model, named MaxGRNet, has been proposed that integrates convolutional inductive biases with multi-axis transformer attention to augment local feature extraction and global contextual modeling. The training and evaluation of all models were done on a workstation with high performance, Google Colab L4.

### 3.4 Explainable AI and quantitative evaluation

To make the model more transparent and reliable, the Explainable Artificial Intelligence (XAI) approaches were employed to learn how the model arrives at a decision. Visual explanations were developed and experimented with through the insertion deletion method. In particular, the check on the accuracy of explanations was conducted by the Area Under the Deletion Curve (AUDC) and Area Under the Insertion Curve (AUIC). The input was used to delete important parts that the explanation map had identified during the deletion process. A sharp decrease in prediction confidence exhibited an excellent explanation. In the insertion process, important parts were added to the input, and a quick rise in confidence showed strong relevance. As shown in Eqs. (1) and (2), lower AUDC values and higher AUIC values mean the explanations are more reliable.

The Area Under the Deletion Curve (AUDC) is defined as:


$$\text{AUDC}=\int_{0}^{1}f(x_{t}^{del})\;dt$$ (1)

Similarly, the Area Under the Insertion Curve (AUIC) is defined as:


$$\text{AUIC}=\int_{0}^{1}f(x_{t}^{ins})\;dt$$ (2)

where $x_{t}^{del}$ represents the image obtained after removing the top *t*% most relevant regions according to *t*he explanation map, and $x_{t}^{ins}$ denotes the image obtained after inserting the top *t*% most relevant regions into a baseline image. The function f(·) represents the model prediction confidence for *t*he target class.

### 3.5 Evaluation strategies

To assess the performance of each of the five models, we used accuracy, sensitivity (or recall), precision (or PPV), and the F1-score as the measures. These metrics were determined by use of true positive (TP), false positive (FP), true negative (TN), and false negative (FN) samples. Accuracy was determined as outlined in Eq. (3), precision in Eq. (4), and recall in Eq. (5). Sensitivity or recall evaluates the capability of a model to recognize all the relevant cases in a data set by the number of true positives divided by the number of false negatives and the total number of true positives. The F1-score, as shown in Eq. (6), is calculated from the ratio of recall and precision.


$$Accuracy=\frac{TP+TN}{TP+FP+TN+FN}$$ (3)

$$Precision=\frac{TP}{TP+FP}$$ (4)

$$Recall=\frac{TP}{TP+FN}$$ (5)

$$F1=\frac{2*Precision*Recall}{Precision+Recall}$$ (6)

In addition, for multi-class ROC analysis, a one-vs-rest (OvR) strategy was employed. For each class, ROC curves were computed against all remaining classes, and macro-averaged AUC values were obtained by averaging the class-wise AUC scores.

## 4. Result analysis

This section provides an in-depth assessment of the MaxGRNet model, comparing it with top baseline architectures. The assessment was based on predictive performance, statistical reliability, and explainability to have a balanced analysis of the model effectiveness. The presentation of all the results was done with standardized evaluation protocols in order to maintain fairness, robustness and reproducibility. Accuracy (Acc), precision, recall, F1-score, and confusion matrices were used to evaluate the performance of the five models using test data. In order to make the experiment robust and reliable, all experiments were performed in a five-fold cross-validation framework where the reported results are the average and standard deviation of the results of the folds. Also, paired statistical significance testing (t-test) was performed to define whether the observed performance differences between the models may be due to significant variation and not to a random variation. This evaluation protocol allows for a fair and rigorous comparison of the proposed model with competing baselines, highlighting both its predictive effectiveness and consistency across multiple data splits.

### 4.1 Predictive performance analysis

Table 5 demonstrates that the proposed MaxGRNet consistently achieves the strongest macro-averaged performance across both datasets, with particularly notable gains on DS1. Specifically, MaxGRNet attains a macro-accuracy of 0.9675 on DS1, outperforming the closest competitor, MaxViT-T (0.9375), by 3.0 percentage points, and exceeding ResNet50 (0.8600) by more than 10 percentage points. Similar improvements are observed for macro-F1 score, with MaxGRNet being 0.9670, which is higher than 0.9370 of MaxViT-T and 0.8600 of ResNet50, which means that the number of improvements in the performance of class-balanced predicting is significant. On DS2, the performance gap between models is smaller overall, which indicates higher variability of datasets. However, MaxGRNet has stable and competitive results with a 0.9290 macro-accuracy and macro-F1 of 0.9284. Although the relative difference to ViT-B16 (0.9300 accuracy, 0.9292 F1-score) is not significant (≤ 0.1 percentage), MaxGRNet demonstrates consistent performance across all metrics, highlighting its robustness rather than reliance on a single evaluation criterion.

**Table 5:** Dataset-wise performance comparison using macro-averaged metrics.

Dataset	Metric	ViT-B16	Swin-T	ResNet50	MaxViT-T	MaxGRNet
DS1	Accuracy	0.8750	0.8950	0.8600	0.9375	**0.9675**
	Precision	0.8780	0.9010	0.8630	0.9410	**0.9670**
	Recall	0.8750	0.8950	0.8600	0.9380	**0.9680**
	F1-score	0.8750	0.8950	0.8600	0.9370	**0.9670**
DS2	Accuracy	0.9300	0.9215	0.9285	0.9135	**0.9290**
	Precision	0.9321	0.9234	0.9302	0.9151	**0.9316**
	Recall	0.9300	0.9215	0.9285	0.9135	**0.9290**
	F1-score	0.9292	0.9206	0.9276	0.9124	**0.9284**

Importantly, MaxGRNet shows metric consistency across accuracy, precision, recall, and F1-score in both datasets, with variations within ±0.001−0.003 on DS1 and ±0.002−0.005 on DS2. This consistency suggests stable decision boundaries and reduced class-wise bias, which is particularly valuable in multi-disease fundus image classification where inter-class visual similarity can adversely affect recall and precision. Overall, the numerical trends in Table 5 indicate that MaxGRNet provides meaningful performance gains on controlled datasets (DS1) while maintaining competitive robustness under more heterogeneous conditions (DS2), thereby adding value beyond incremental accuracy improvements.

**Fig 6:**
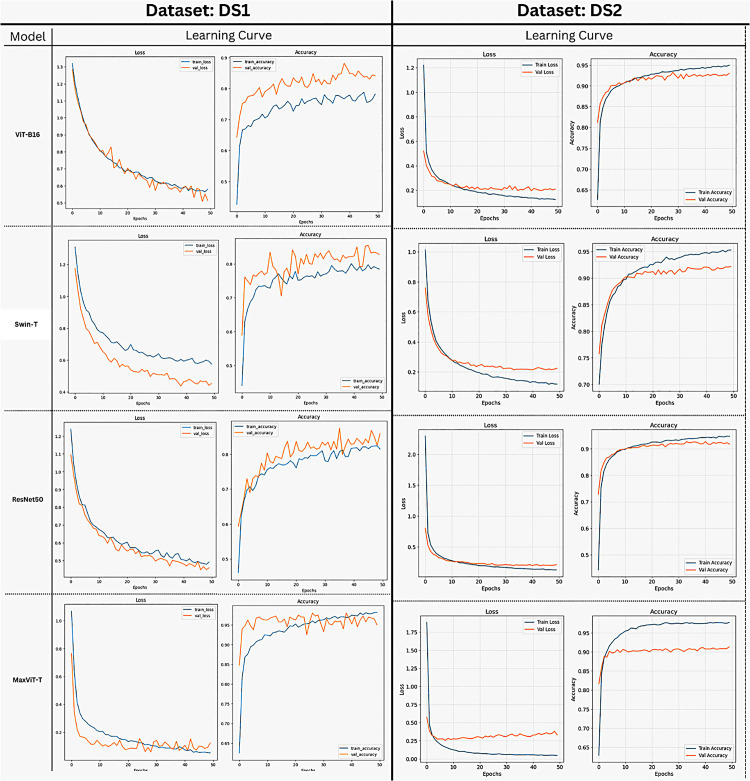
Accuracy curves for transfer learning-based models used in our study.

The accuracy trends illustrated in Fig 6 indicate that Swin-T consistently outperformed ViT-B16 and ResNet50 across training epochs, achieving a higher and more stable convergence with an average test accuracy of 0.8950, compared to 0.8750 for ViT-B16 and 0.8600 for ResNet50. The learning curve of Swin-T shows rapid performance gains in the early epochs, followed by stable saturation, suggesting improved optimization behavior and stronger generalization. Although ViT-B16 demonstrates competitive learning dynamics, it converges to a lower accuracy level, whereas ResNet50 exhibits comparatively slower convergence and reduced final performance. These observations are consistent with the quantitative results reported in Table 5, reinforcing the superior classification capability of Swin-T compared to the baseline transfer learning models.

**Fig 7:**
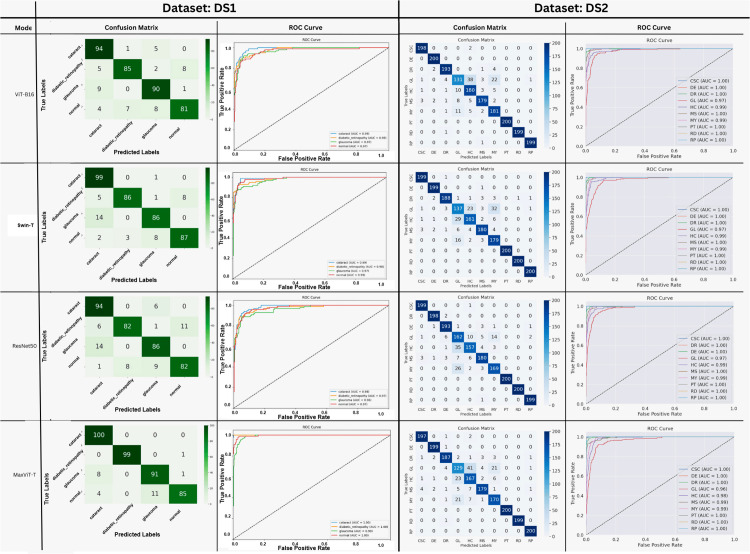
Confusion matrices and class-wise ROC curves for ViT-B16, Swin-T, ResNet50, and MaxViT-T on DS1 and DS2.

Fig 7 presents the confusion matrices and class-wise ROC curves for ViT-B16, Swin-T, ResNet50, and MaxViT-T evaluated on DS1 and DS2. On DS1, transformer-based models show strong diagonal dominance, with MaxViT-T correctly classifying up to 100 cataract samples and 99 diabetic retinopathy samples, while ResNet50 exhibits higher misclassification, including up to 14 glaucoma samples. The corresponding ROC curves indicate high discriminative capability, with class-wise AUC values in the range of 0.99−1.00.

For DS2, which contains a larger number of disease classes, all models maintain high true positive rates, with most classes achieving AUC values of ≥0.97. Despite increased class complexity, the confusion matrices demonstrate that the majority of samples are correctly classified along the diagonal, with several classes exceeding 180 correct predictions. These results confirm consistent class-wise separability across both datasets.

**Fig 8:**
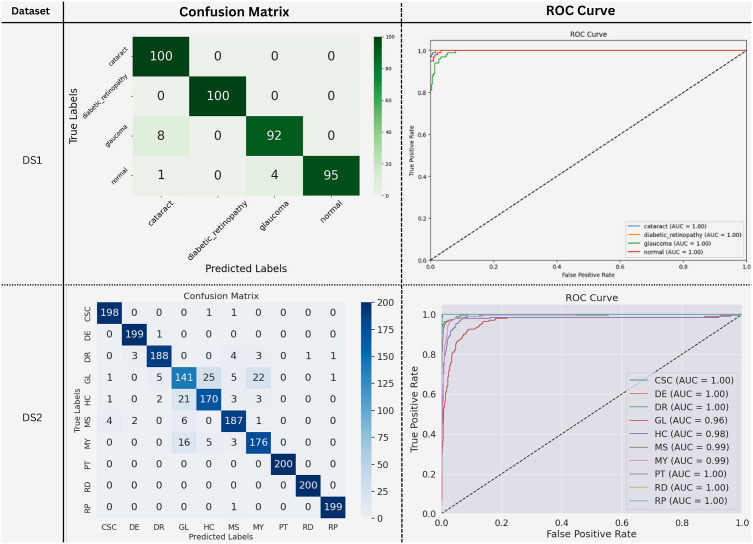
Confusion matrices and class-wise ROC curves for MaxGRNet on DS1 and DS2.

Fig 8 presents the confusion matrices and class-wise ROC curves for the proposed MaxGRNet model evaluated on DS1 and DS2. On DS1, MaxGRNet achieves near-perfect classification, correctly identifying 100 cataract and 100 diabetic retinopathy samples, while 92 glaucoma and 95 normal images are correctly classified. Limited misclassifications are observed primarily between glaucoma and visually similar classes (e.g., 8 glaucoma samples misclassified as cataract). The corresponding ROC curves indicate excellent discriminative performance, with all classes achieving an AUC of 1.00.

On DS2, which includes a larger number of disease categories, the confusion matrix exhibits strong diagonal dominance, with several classes such as PT, RD, and RP achieving 200 correct predictions. Other classes, including CSC (198), DE (199), and DR (188), also demonstrate high true-positive counts, while increased confusion is observed among visually overlapping categories such as GL, HC, and MY. The ROC analysis confirms robust class-wise separability, with most classes attaining AUC values in the range of 0.99−1.00, and the lowest observed AUC being 0.96 for GL.

### 4.2 Cross-validation and statistical significance analysis

To assess the stability and statistical reliability of the model performance, five-fold cross-validation was conducted on DS1, and the results are summarized in Table 6. Among the baseline models, ResNet50 and MaxViT-T achieved competitive results, with mean accuracies of 0.9419 ± 0.0134 and 0.9406 ± 0.0129, respectively, indicating a stable generalization across folds. ViT-B16 and Swin-T exhibited slightly lower but consistent performance, reflecting higher variability across the folds. Notably, the proposed MaxGRNet outperformed all competing models, achieving the highest accuracy (0.9494±0.0106), along with superior precision, recall, and F1-score. The reduced standard deviation further indicates more stable and statistically meaningful performance gains, demonstrating the robustness and reliability of the MaxGRNet under cross-validation settings. The paired statistical comparison of the proposed MaxGRNet and other baselines is presented in Table 7. From there, it has been found that the proposed MaxGRNet has better performance gain over other attention-based baselines.

**Table 6:** Cross-validation performance comparison of different models on DS1. Values are reported as mean ± standard deviation across 5 folds.

Model	Accuracy	Precision	Recall	F1-score
Swin-T	0.9319±0.0115	0.9326±0.0119	0.9319±0.0115	0.9319±0.0117
MaxViT-T	0.9406±0.0129	0.9412±0.0126	0.9406±0.0129	0.9405±0.0128
ResNet50	0.9419±0.0134	0.9425±0.0138	0.9419±0.0134	0.9419±0.0135
ViT-B16	0.9343±0.0123	0.9349±0.0128	0.9343±0.0123	0.9342±0.0125
**MaxGRNet (Proposed)**	0.9494±0.0106	0.9497±0.0105	0.9494±0.0106	0.9494±0.0108

**Table 7:** Paired statistical significance analysis (5-fold cross-validation) comparing MaxGRNet with baseline models on DS1. Δ denotes the mean fold-wise difference (MaxGRNet-Baseline). 95% confidence intervals (CI) are computed over paired fold-wise differences using the *t*-distribution (*df* = 4). *p*_Holm_ denotes the Holm–Bonferroni corrected p-values across the four baseline comparisons (paired t-test). *p*_Wilc._ denotes Wilcoxon signed-rank test p-values (two-sided).

Baseline	Δ Acc	95% CI (Acc)	*p*_Holm_	*p*_Wilc._	Δ F1	95% CI (F1)	*p*_Holm_	*p*_Wilc._
MaxViT-T	+0.0089	[−0.0034, +0.0212]	0.2317	0.0679	+0.0089	[−0.0034, +0.0212]	0.2321	0.0625
ViT-B16	+0.0151	[+0.0054, +0.0248]	0.0374	0.0625	+0.0152	[+0.0051, +0.0252]	0.0414	0.0625
Swin-T	+0.0175	[+0.0074, +0.0276]	0.0345	0.0625	+0.0175	[+0.0071, +0.0279]	0.0380	0.0625
ResNet50	+0.0075	[−0.0147, +0.0297]	0.4010	0.6250	+0.0075	[−0.0148, +0.0297]	0.4035	0.4375

### 4.3 Explainability and faithfulness evaluation

Beyond predictive performance, the faithfulness of the model explanations was quantitatively evaluated using insertion and deletion metrics. Table 8 reflects a qualitative comparison where results are reported as mean AUC values with 95% bootstrap confidence intervals. Based on the insertion and deletion behavior, it shows that MaxGRNet is superior in explanation. The prediction confidence decreases significantly when the highlighted regions are deleted, indicating faithful explanations.

**Table 8:** Quantitative evaluation of explainability using insertion/deletion AUC on the test set under a GaussianBlur baseline (kernel = 51, σ=8.0, 50 steps). We report mean AUC with 95% bootstrap confidence intervals. Higher Insertion and higher (1- deletion) indicate more faithful explanations. Correct-only protocol was used (target class = predicted class).

Model	Images	Insertion AUC ↑	Deletion AUC ↓	1-Deletion AUC ↑
MaxGRNet	379	0.661 (0.631–0.690)	0.528 (0.490–0.566)	0.472 (0.434–0.510)
MaxViT-T	377	0.619 (0.592–0.644)	0.562 (0.535–0.588)	0.438 (0.412–0.465)
ResNet50	384	0.705 (0.682–0.728)	0.611 (0.583–0.640)	0.389 (0.360–0.417)

Then the cardinal target is to utilize a common subset of images where the proposed MaxGRNet outperforms MaxViT-T in the case of insertion and 1-deletion AUC. Table 9 shows that compared to ResNet50, MaxGRNet has a lower insertion AUC. This implies a lower confidence recovery. In contrast, the deletion AUC is higher, which denotes more reliable explanations, especially in terms of feature necessity.

**Table 9:** Paired statistical comparison of explanation faithfulness using per-image AUCs on the common subset (intersection by index, *n* = 369). We report the mean difference Δ (MaxGRNet minus baseline model) and p-values from paired t-test and Wilcoxon signed-rank test. A positive Δ indicates that MaxGRNet is better for Insertion and (1-Deletion). GaussianBlur baseline (k = 51, σ=8.0; 50 steps).

Comparison	Metric	Δ	t-test p	Wilcoxon p
MaxGRNet vs MaxViT-T	Insertion AUC ↑	+0.0436	1.999e–07	4.208e–09
MaxGRNet vs MaxViT-T	1-Deletion AUC ↑	+0.0358	0.002139	0.01444
MaxGRNet vs ResNet50	Insertion AUC ↑	–0.0425	3.913e–05	0.0002604
MaxGRNet vs ResNet50	1-Deletion AUC ↑	+0.0860	1.647e–12	6.002e–13

The consistent superior prediction accuracy is shown in Table 10, where MaxGRNet provides better explainability than MaxViT-T. Even other architectures, such as Swin-T and ViT-B, underperformed compared to MaxGRNet.

**Table 10:** Overall statistical significance summary for MaxGRNet vs baselines on DS1. Prediction: 5-fold paired tests with Holm correction. Explainability: per-image paired tests on common test images (GaussianBlur baseline, 50 steps; correct-only protocol), using Insertion AUC and 1–Deletion AUC.

Baseline	Significant?	Why / Why not (based on p-values)	Evaluation
MaxViT-T	**Yes (XAI)**	**Explainability is significant**: MaxGRNet improves Insertion AUC ($p_{t}\approx 2.0\times 10^{-7}$, $p_{wil}\approx 4.2\times 10^{-9}$) and 1–Deletion AUC ($p_{t}\approx 0.0021$, $p_{wil}\approx 0.014$). **Prediction is not significant**: CV gains are small and not significant after Holm correction (*p*_holm_ > 0.05).	Prediction (5-fold CV) + Explainability (Insertion/1–Deletion)
ResNet50	**Mixed (XAI)**	**Explainability is mixed**: MaxGRNet is worse on Insertion (negative Δ, significant) but better on 1–Deletion (positive Δ, highly significant: $p_{t}\approx 1.65\times 10^{-12}$, $p_{wil}\approx 6.0\times 10^{-13}$). **Prediction is not significant**: CV difference not significant after Holm correction (*p*_holm_ > 0.05).	Prediction (5-fold CV) + Explainability (Insertion/1–Deletion)
Swin-T	**Yes (Prediction)**	**Prediction is significant**: MaxGRNet significantly improves Accuracy/F1 after Holm correction (*p*_holm_ < 0.05). The explainability comparison is not reported in the current XAI tables.	Prediction (5-fold CV)
ViT-B16	**Yes (Prediction)**	**Prediction is significant**: MaxGRNet significantly improves Accuracy/F1 after Holm correction (*p*_holm_ < 0.05). Explainability comparison not reported in the current XAI tables.	Prediction (5-fold CV)

**Fig 9:**
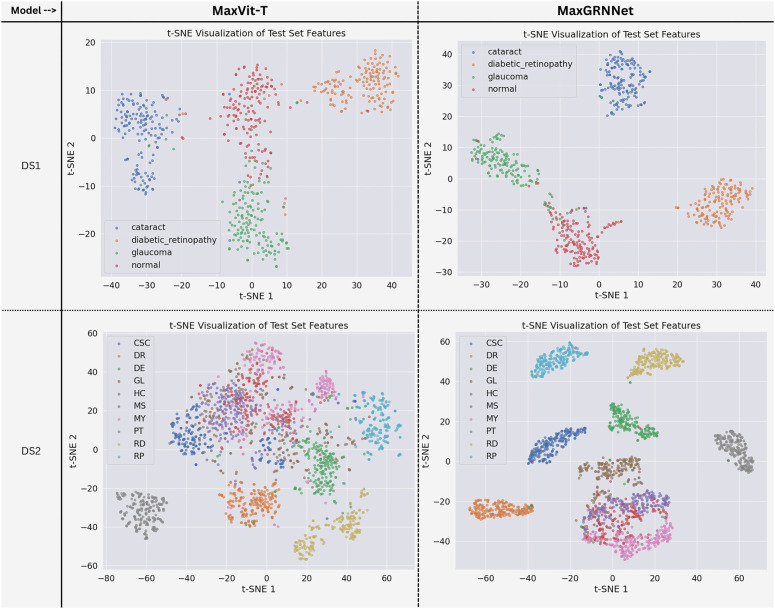
t-SNE Feature separation plot for MaxViT-T and proposed MaxGRNet model with the test set of both datasets.

The t-SNE visualizations in Fig 9 demonstrate clear differences in feature discriminability between MaxViT-T and the proposed MaxGRNet on both DS1 and DS2 datasets. While MaxViT-T exhibits partially overlapping clusters, particularly among visually similar classes, MaxGRNet produces more compact intra-class distributions and larger inter-class margins, indicating an improved feature separability. The enhanced cluster compactness and margin maximization observed with MaxGRNet reflect its ability to learn more discriminative representations. These qualitative findings are consistent with the superior quantitative performance of MaxGRNet in terms of accuracy, F1-score, and AUC metrics.

**Fig 10:**
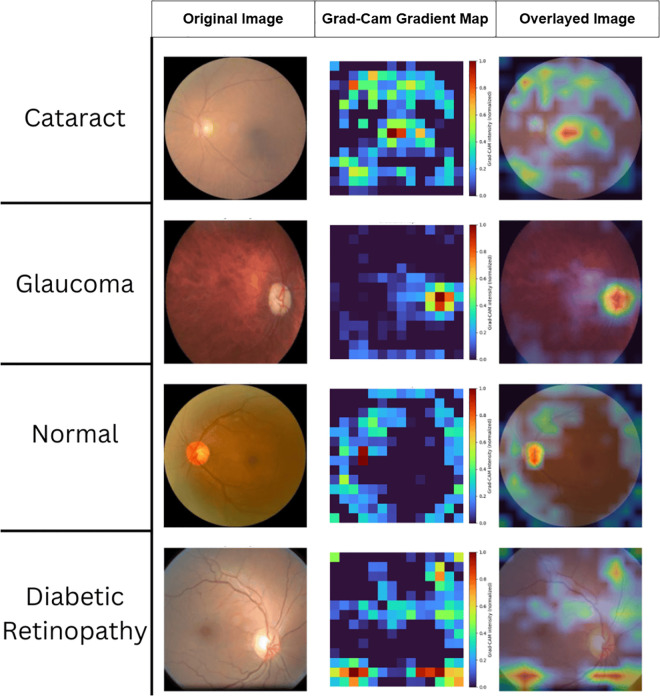
Visualizing the proposed MaxGRNet model with gradient map.

Gradient-weighted Class Activation Mapping (Grad-CAM) is used to show in Fig 10 the important parts of the input images that have a significant impact on the model’s decision-making regarding classification [32]. Using the MaxGRNet model layers designs, Grad-CAM maps were created by determining the gradient of the expected class score for the convolutional feature maps from the last convolutional layer.

MaxGRNet was selected for explainable artificial intelligence (XAI) to demonstrate its capability in extracting features from fundus images and interpreting the decision-making process for each class. The attention maps that were created placed the highlighted areas on top of the original images, making it easy to see what the model was thinking about when it made its choice. This shows how the attention maps can find important parts of the images; the red and yellow areas show the most important features that make a significant difference in the model’s predictions. The red areas show the most activity, the green areas show modest contributions, and the blue areas show that they have almost no effect on the model output. These images are crucial for understanding the interpretability and reliability of the model because they show that the model focuses on the right areas for grouping. By showing pictures of what the model predicts will happen, the methods used make things clearer and more trustworthy when they are used in real medical settings.

**Fig 11:**
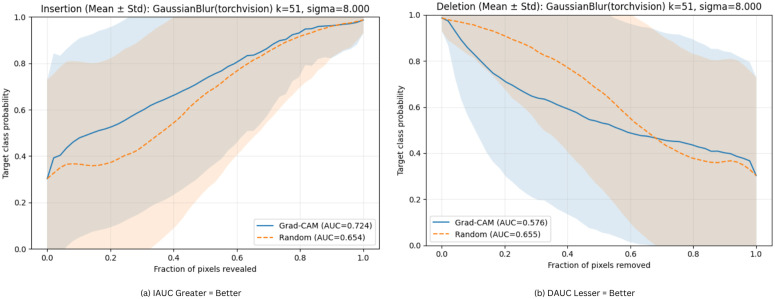
Insertion and deletion curves (mean ± std) comparing Grad-CAM vs random mask under a GaussianBlur baseline (*k* = 51, α=8) for proposed MaxGRNet.

The overall insertion and deletion curves are shown in Fig 11. The curves were obtained using a standard Gaussian blur baseline. For the random baseline, Grad-CAM achieved a higher AUC. It is understood that the revealed regions effectively recover the target class probability. In the deletion plot, the AUC value is opposite that is lower. Therefore, the confidence degraded rapidly when the highlighted regions were removed. This represents greater faithfulness and meaningful explanations.

**Fig 12:**
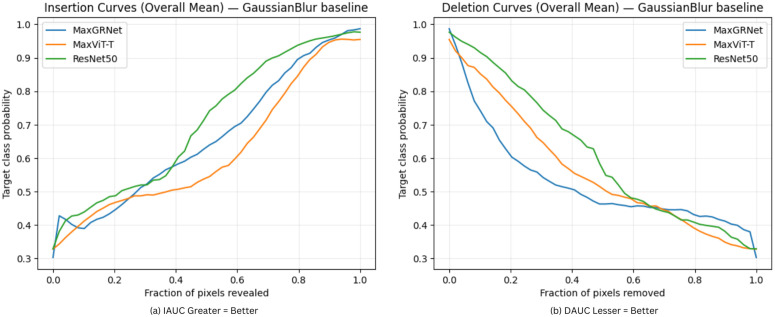
Overall insertion and deletion curves (mean) under a GaussianBlur baseline comparing MaxGRNet, MaxViT-T, and ResNet50.

**Fig 13:**
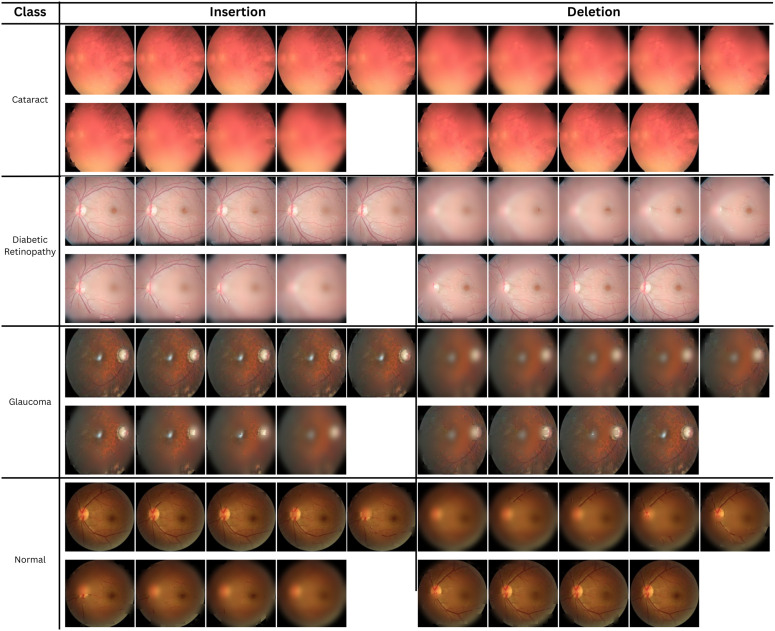
Examples of images passed to the model during the computation of IAUC and DAUC with Grad-CAM, per class.

Furthermore, the overall mean insertion and deletion under GaussianBlur by comparing MaxGRNet, MaxViT-T and ResNet50 is shown in Fig 12. Here, the supremacy of MaxGRNet over the other architectures can be observed. Fig 13 shows some of the images that were passed during the computation of the IAUC and DAUC with Grad-CAM. In different phases, the insertion and deletion processes were better in MaxGRNet than in the other architectures.

## 5. Discussion

The Vision Transformer’s multi-axis attention mechanism enables effective modeling of complex spatial and contextual patterns in fundus images, making it highly suitable for discriminative feature extraction required for accurate ophthalmic disease classification. Building on this capability, the MaxViT architecture enhances the understanding of both local and global dependencies, surpassing conventional CNNs and earlier transformer-based models in capturing subtle disease-specific visual cues. This capability supports improved discrimination between retinal conditions that may share certain overlapping visual characteristics in fundus images. However, clinical diagnosis of these diseases requires comprehensive ophthalmic assessment beyond image-based analysis.

The proposed MaxGRNet also extends this paradigm with the MaxViT combined with a Global Response Normalization (GRN)-based MLP that enables the model to highlight the diagnostically relevant features, whilst minimizing the irrelevant activations. This design improves class separability and enables the detection of fine-grained pathological patterns that are often overlooked by standard deep learning approaches. Moreover, MaxGRNet was computationally efficient in that it performs well without overly consuming resources, which increases its prospective application to clinical decision-support and research-oriented applications, as long as it can be validated in the future externally.

The overall results in Table 11 show that the proposed MaxGRNet has a better F1-score as compared to the state-of-the-art methods presented in the literature in several datasets. These results show how useful, well-scaling, and strong the proposed framework is, which contributes to the fact that it can be a noninvasive computational analysis framework suitable for research and benchmarking of ophthalmic image analysis. Upon additional confirmation, MaxGRNet may make an exceptionally positive contribution to the development of AI-based personalized healthcare solutions.

**Table 11:** Comparison between the proposed MaxGRNet and the models from literature in terms of F1-Score.

Literature	Proposed Architectures	Dataset	F1-Score
Chea et al. [18]	SEnet with HRnet as Backbone	OIA-ODIR	88.56
Gu et al. [14]	Vision Transformer and Residual Attention (ViT+CSRA)	DDR dataset and IDRiD	91.54
Pan et al. [22]	InceptionV3 and ResNet50	Private	93.81, 91.76
Gu et al. [23]	Hybrid Convolution and Vision transformer based model	OCTID	94.92
Liu et al. [33]	CNN-Trans: a hybrid CNN with attention mechanism and feature fusion	ODIR-5 K, eye_disease _classification	80.68, 94.72
Babaqi et al. [34]	EfficientNet CNN	eye_disease_classification	94
Biswas et al. [35]	CNN with (Rectified Linear Unit) ReLU	eye_disease_classification	93
Darji et al. [36]	VGG-16, and MobileNet	eye_disease _classification	84.83, 88.15
Rajalakshmi et al. [37]	EfficientNetB3 CNN	eye_disease_classification	93
Zannah et al. [38]	BayeSVM500: combining Bayesian methods and SVM	eye_disease_classification and combined with others.	95.90
Elkenawy et al. [39]	AlexNet	eye_disease_classification	93.65
**This research**	**MaxGRNet: Combining MaxViT and GRN MLP layers**	**eye_disease_classification**	**96.75**

Despite the fact that the proposed MaxGRNet model shows higher classification performance, it is not as parameter-efficient as some of the lightweight baseline models are. The main goal of this work is to achieve a high representational ability and strength instead of reducing the number of parameters and deployment-level latency. Accordingly, computational efficiency is discussed in terms of architectural design choices and performance–complexity trade-offs, rather than absolute runtime or memory optimization. Future directions would involve model compression, pruning and lightweight versions of transformers to achieve efficiency in deployment without compromising in predictive performance.

## 6. Conclusion and future works

This study presents an effective framework for eye disease classification using a multi-axis vision transformer (MaxViT) enhanced with Global Response Normalization-based MLP layers applied to color fundus images. By leveraging advanced transformer-based feature extraction and Explainable Artificial Intelligence (XAI) techniques, the proposed approach improves classification performance and model interpretability compared to conventional CNN-based architectures. The proposed MaxGRNet model achieved an accuracy of 96.75% on the eye_diseases_classification dataset, demonstrating its ability to distinguish between multiple retinal conditions, including cataracts, diabetic retinopathy, and glaucoma. Despite these encouraging results, some misclassification was observed for glaucoma cases, indicating challenges associated with subtle inter-class visual variations and dataset complexity. This highlights the inherent difficulty of achieving uniformly high performance across all disease categories, particularly when working with limited and heterogeneous datasets. Future work will focus on addressing these limitations through targeted architectural refinements, the incorporation of larger and more diverse datasets, and the exploration of advanced data augmentation and regularization strategies to further enhance generalization. The proposed MaxGRNet framework addresses several practical challenges in medical image analysis, such as data scarcity and computational efficiency, making it well suited for scalable experimental and translational research settings. While the current study is limited to technical performance evaluation, the integration of XAI provides valuable insights into the model’s decision-making process, supporting transparency and interpretability in ophthalmic image analysis. Future studies should investigate external validation across multi-center datasets and assess the applicability of the proposed framework in real-world decision-support scenarios. Overall, this work provides a robust methodological foundation that may facilitate future research and development in AI-assisted ophthalmic image analysis and related medical imaging applications.
